# Sleep Quality, Mental and Physical Health: A Differential Relationship

**DOI:** 10.3390/ijerph18020460

**Published:** 2021-01-08

**Authors:** Violeta Clement-Carbonell, Irene Portilla-Tamarit, María Rubio-Aparicio, Juan J Madrid-Valero

**Affiliations:** Department of Health Psychology, Faculty of Health Science, University of Alicante, Carretera de San Vicente del Raspeig s/n, 03690 Alicante, Spain; violeta.clement@ua.es (V.C.-C.); irene.portilla@ua.es (I.P.-T.); maria.rubio@ua.es (M.R.-A.)

**Keywords:** mental health, physical health, Pittsburgh Sleep Quality Index, quality of life, sleep quality

## Abstract

This study aimed to explore the association between sleep quality and its components and both dimensions of health-related quality of life (HRQoL) in a sample of young adults. The sample comprised 337 participants with a mean age of 19.6 y (SD = 2.22). Sleep quality and HRQoL were measured through the Pittsburgh Sleep Quality Index and the SF-12, respectively. Regression analyses were used to investigate the association between sleep quality and HRQoL. Our results confirm the significant association between sleep quality and both physical (*p* = 0.015; β = −0.138; R^2^ = 0.07) and mental (*p* < 0.001; β = −0.348; R^2^ = 0.22) HRQoL in the adjusted models. However, our results also highlight the differential association between sleep quality and mental and physical HRQoL. Whereas all the sleep quality components (except sleep latency; *p* = 0.349) were significantly associated with mental HRQoL (*p* < 0.05), just two subscales (subjective sleep quality; *p* = 0.021; β = −0.143 and sleep disturbances *p* = 0.002; β = −0.165) showed a significant association. This study showed that there is a stronger association between sleep quality and mental health than sleep quality and physical health in young adults.

## 1. Introduction

Sleep is an essential and universal function for humans [1]. Sleep is now considered one of the three basic pillars of health together with diet and exercise [2]. Poor sleep quality has a negative impact in different areas related to physical health such as type 2 diabetes [3], hypertension [4], chronic pain [5] and higher levels of body mass index [6,7] among other adverse consequences.

In addition to this, poor sleep quality is also related to negative psychological consequences such as anxiety and depression [8], aggression [9], altered cognitive functioning [10], and attention-deficit/hyperactivity disorder [11] among others.

The above-cited literature shows the importance of sleep quality for both physical and mental health. Adolescence is a critical period for sleep quality [12] and is also critical for psychopathology [13]. The scientific literature reveals that adolescence is a stage of the lifespan with a high vulnerability. There is a high comorbidity between psychiatric disorders and sleep problems [14,15]. This is especially important should we consider that there is a bi-directional relationship between sleep disturbances and mental health [8,14]. Previous research has shown that sleep treatments may have a positive impact not only on sleep outcomes but also on mental health and HRQoL (Health-Related Quality of Life) [14,16,17]. Researching the relationship between sleep quality and HRQoL in adolescence and young adulthood might put us in a stronger position to prevent the development (or worsening) of both sleep and health problems since sleep is a modifiable determinant of health [18].

Sleep quality is a broad concept that includes several measures such as sleep latency, sleep efficiency, sleep disturbances, sleep duration, and use of sleeping medication among others [19]. Previous research has shown that poor sleep quality among university students is high [20,21,22] There is a significant amount of scientific literature that has previously addressed the relationship between sleep quality and HRQoL in adolescents [23] and young adults [24,25]. It has been found that people suffering from sleep disorders have lower levels of both physical and mental HRQoL showing moderate R^2^ for the physical and mental components, although depending on the disorder, the impact of some disorders is more pronounced on the mental component or the physical component (e.g., apnea has a higher impact on physical health whereas insomnia has a higher impact on mental health) [26]. In addition to this, a study performed with a sample of children showed that distinct sleep quality subtypes could have a differential impact on HRQoL [27].

Even though sleep quality is a broad concept that has a significant impact on both mental and physical HRQoL, there is a scarcity of research addressing this issue. Therefore, this study was aimed to disentangle the role of the specific components of sleep quality on both physical and mental HRQoL in a sample of young adults controlling for relevant confounders.

## 2. Materials and Methods

### 2.1. Participants

All participants were university students from the University of Alicante. The University of Alicante is a public University in the south-east of Spain. Data were collected through an online interview and promoted using email distribution lists and university virtual platforms, during the third term of 2019. No exclusion criteria were applied. However, participants above 30 y (7.67%) were excluded because our target group was young adults and to maintain the sample homogeneously. The final sample comprised 337 participants over 18 y, all of whom provided informed consent. The sample was 78.6% female and the mean age was 19.63 y (SD = 2.22).

### 2.2. Measures

Sleep quality was measured by means of the Pittsburgh Sleep Quality Index (PSQI). The PSQI is a widely used questionnaire [19] This questionnaire provides a global score and 7 sub-scales. These seven sub-scales (i.e., subjective sleep quality, sleep latency, sleep duration, sleep efficiency, sleep disturbances, use of sleeping medication and daytime dysfunction) build the global score which ranges from 0 to 21 (higher scores represent poorer sleep quality).

The Spanish validated version of the PSQI was used in this sample [28]. This questionnaire has also shown adequate psychometric properties [28]. To check the internal consistency of this scale, in the current sample, which is how closely a set of items are as a group we calculated Cronbach’s Alpha and the result was 0.67 for the global score.

HRQoL was measured using the SF-12 Health Survey (SF-12) which has been validated in a Spanish population [29]. This is an instrument that evaluates the quality of life in the general population. It comprises 12 items and has 8 dimensions (i.e., general health, physical functioning, role physical, role emotional, body pain, social functioning, mental health, and vitality). These dimensions yield two summary components: the physical component summary (PCS) and the mental component summary (MCS). These two components range from 1 (“worse health condition”) to 100 (“best health condition”). Scores equal to or less than 30 are considered a risk. The SF-12 is a valid and reliable measurement tool, with an internal consistency exceeding 0.70 [25]. In the current sample, the Cronbach’s Alpha was 0.81 for the global score.

Diet, stress and exercise were measured using the Lifestyle Indicator questionnaire. The diet component ranges from 0 to 15, exercise from 0 to 24 and stress from 1 to 6. Higher scores represent healthy eating habits, higher levels of exercise and low levels of stress [30].

### 2.3. Statistical Analyses

All the analyses were performed using STATA statistical software (v12.0). We performed descriptive statistics for demographics and the key variables involved in this study, sex differences were tested. Both dimensions of HRQoL (i.e., PCS, MCS) were treated as continuous variables and as was the global PSQI. As for sleep quality sub-scales, they were treated as continuous variables when it was possible (i.e., sleep latency, sleep duration, sleep efficiency and sleep disturbances). The rest of the sub-scales were treated as ordinal variables according to the original coding from the PSQI. Sleep latency was measured in minutes, sleep duration in hours and sleep efficiency was calculated following the instructions from the PSQI questionnaire to get the % of sleep efficiency. Finally, sleep disturbances were calculated by the sum of all the possible sleep disturbances presented in the questionnaire and their frequency (i.e., 0: not during the past month; 1: less than once a week; 2: once or twice a week; 3: three or more times a week). We considered age, sex, diet, stress and exercise as covariates. These variables showed an association of *p* < 0.20 in the univariate model between HRQoL and the covariate. Regression models were applied to test the association between each sub-scale of the PSQI and also the global score. Therefore, sixteen independent regressions models were fitted (one for each PSQI subscale and one for the global score) where HRQoL (PCS or MCS) was the outcome. All these models were fitted adjusted and unadjusted (i.e., Pearson’s correlation between the sleep variable and the HRQoL component) for covariates (age, sex, diet, exercise and stress).

As diet, stress and exercise have shown to be associated with both sleep quality and HRQoL moderation analyses were also performed to investigate if the association between sleep quality and PCS/MCS was moderated by one of these proposed covariates. Therefore, all models were also tested using these covariates as moderators.

Our sample showed 80% of the power to detect correlations above 0.15.

## 3. Results

### 3.1. Descriptive Statistics

Table 1 shows the descriptive statistics. Regarding sleep quality, the mean value for the global score was 7.03 (SD = 3.32). As for the sub-scales, the average sleep latency was 31.62 min (SD = 28.27) and the mean sleep time was 6.88 h (SD = 1.39). The mean values for the SF-12 subscales were 53.28 (SD = 6.94) and 39.01 (SD = 13.42) for physical and mental HRQoL, respectively. No sex differences were found for Sleep quality nor HRQoL. Regarding the covariates, age and stress showed statistically significant differences between men and women (*p* < 0.05).

### 3.2. Association between Sleep Quality and Physical Health-Related Quality of Life

A significant relationship between global PSQI score and physical HRQoL was found in both models, unadjusted (*p* = 0.002; β = −0.172) and adjusted for covariates (*p* = 0.015; β = −0.138) the R^2^ values were 0.03 and 0.07 for the unadjusted and adjusted models respectively.

However, when this relationship was analyzed at the scale level, only two subscales presented a significant association with physical HRQoL. These two subscales were subjective sleep quality (*p* = 0.021; β = −0.143) and sleep disturbances (*p* = 0.002; β = −0.165) with R^2^ values of 0.07 for both variables. The rest of the subscales reached the level of significance, neither in the adjusted model nor in the unadjusted model (Table 2).

### 3.3. Association between Sleep Quality and Mental Health-Related Quality of Life

Global PSQI score also presented a significant association with mental HRQoL unadjusted (*p* < 0.001; β = −0.410) and adjusted model (*p* < 0.001; β = −0.348) the R^2^ values were 0.17 and 0.22 for the unadjusted and adjusted models respectively. Interestingly, all the PSQI subscales, except sleep latency (*p* = 0.349), showed a significant association (*p* < 0.050) with mental HRQoL with beta values ranging from 0.129 to 0.130 for sleep duration and sleep efficiency, respectively, and ranging from −0.143 to −0.398 for subjective sleep quality, sleep disturbances, use of sleeping medication and daytime dysfunction (Table 3). R^2^ values ranged from 0.11 to 0.25.

The relationship between sleep quality and PCS/MCS was not moderated by any of the tested covariates (stress, diet, exercise) (*p* > 0.05). Furthermore, moderation models were fitted for all the PSQI subscales but again none of these variables moderated the relationship between sleep quality subscales and PCS/MCS (*p* > 0.05).

## 4. Discussion

This study started with the aim of deepening knowledge about the relationship between sleep quality and HRQoL. Our results confirm the significant association between sleep quality and both dimensions of HRQoL. These results match well with the previous literature where significant associations have also been found [24,26,31], although lower R^2^ values were found. This could be due to differences related to sample characteristics. Furthermore, this study has also thoroughly examined this relationship to elucidate the underpinnings of it. Our findings indicate a differential association between the different components of sleep quality and the two studied components of HRQoL (i.e., PCS and MCS). Global sleep quality was significantly related to both components of HRQoL. It is important to note that this relationship was not significantly moderated by stress, diet or exercise, variables that are robustly linked to both sleep quality and HRQoL. However, whereas all the PSQI subscales were significantly associated with MCS, except sleep latency, just two subscales were significantly associated with PCS (i.e., subjective sleep quality and sleep disturbances). Therefore, these results reveal that the impact of each component of sleep quality is different for both constructs of HRQoL. Indeed, it seems that the impact of poor sleep quality is more harmful to MCS than for PCS. However, the comparison with other studies is difficult due to the scarcity thereof. Similar results were found in a study with a sample of young adults where some symptoms of insomnia (i.e., trouble falling asleep, sleep latency ≥30 min and daytime sleepiness) were significantly associated with poor mental HRQoL but not with poor physical HRQoL [24]. Conversely, in another study that used the PSQI, significant correlations were found between both physical and psychological domains [32] In addition to this, a recent study carried out in a sample of children aged between 9 and 11 y showed a significant association between HRQoL and self-reported quality and quantity of sleep but no association was established with device-measured total sleep time, sleep timing or sleep efficiency [33]

Our results are also in line with previous research that has shown that some sleep disorders are more related to PCM (e.g., sleep apnea) whereas others are more related to MCS (e.g., insomnia) [26]. Mechanisms underlying this relationship are diverse and complex. For example, poor sleep quality has been linked with a higher BMI where it seems that poor sleep quality leads to an increase in BMI [7]. Sleep quality may affect BMI through hormonal and biochemical changes such as variations in leptin, ghrelin and cortisol levels or increased resistance to insulin [34]. Some mechanisms underlie the relationship between poor sleep quality and depression and anxiety. For example, the corticolimbic circuitry seems to be affected by poor sleep quality which is related to difficulties in affective reactivity and regulation [35]. Furthermore, the reward-brain related function may be disrupted by poor sleep quality [36] Those are just a few examples of how poor sleep quality could affect HRQoL. However, an exhaustive review of all the mechanisms underlying these relationships would be out of the scope of this study and require an extensive explanation.

Levels of MCS and PCM are similar to those obtained with similar samples [37]. Levels of poor sleep quality are in line with previous research (in the upper end) which shows that poor sleep quality is high among university students [20,21,22]. This reveals that poor sleep quality is high among adolescents and young adults. This might be due to biological changes associated with adolescence but also social factors [38] As it has been stated before, adolescence is a critical period for both mental and physical health. It is possible that in our sample the consequences of poor sleep are more notorious in the MCS component since our sample is quite young and physical consequences due to poor sleep may have not appeared yet because they need more time to manifest. This study shed some light on the pattern of relationships between HRQoL and sleep quality. Prevention programs should be applied in the early stages to prevent HRQoL problems and also sleep problems. Further research is needed to confirm these findings in other samples and using longitudinal designs.

### Strengths and Limitations

This study has several strengths such as the use of a young adult sample which allows us to study the relationship between HRQoL and sleep quality in a life-span stage where both problems are common. The use of the PSQI questionnaire allowed us to study the impact not only of sleep quality in general but also on each specific component. However, this study is not free of limitations. First of all, our measures were self-reported, but these questionnaires are widely used to measure sleep quality and HRQoL. Another limitation that should be mentioned is the representativeness of the sample. The sample was recruited using the university resources and that could have resulted in a bias of our results. For example, there could be differences between participating and non-participating students. Finally, several confounders were not taken into account such as BMI, socioeconomic status or mental and physical disorders which could affect our results. Therefore, our results must be interpreted considering these limitations and the generalizability of the results is not completely guaranteed. Future studies should address this issue to extend these findings to different populations

## 5. Conclusions

Our results confirm the significant association between HRQoL and sleep quality even controlling for relevant covariates (i.e., diet, stress and exercise). Our results also show the differential association between sleep quality and MCS and PCS since just two sleep quality subscales were significantly associated with PCS whereas most of them were associated with MCS. Indeed, the impact of poor sleep quality is larger on the MCS component than on the PCS component. This study highlights the importance of considering sleep quality as a multicomponent measure and also could help us develop more specific programs to prevent both sleep problems and HRQoL problems.

## Figures and Tables

**Table 1:** Descriptive statistics.

	Male	Female	Total
N (%)	72 (21.4)	265 (78.6)	337 (100)
		Mean (SD)	
Age (SD) *	19.11 (1.67)	19.77 (2.33)	19.63 (2.22)
PSQI Global Score	6.69 (2.85)	7.11 (3.43)	7.03 (3.32)
Subjective sleep quality	1.10 (0.67)	1.28 (0.72)	1.24 (0.71)
Sleep latency (in minutes)	34.93 (27.96)	30.72 (28.34)	31.62 (28.27)
Sleep duration (in hours)	6.82 (1.40)	6.89 (1.40)	6.88 (1.39)
Sleep efficiency (%)	86.87 (22.00)	82.36 (19.68)	83.31 (20.40)
Sleep disturbances	5.83 (3.14)	6.62 (3.64)	6.45 (3.55)
Use of sleeping medication	0.28 (0.68)	0.31 (0.76)	0.30 (0.74)
Daytime dysfunction	1.13 (0.80)	1.32 (0.83)	1.28 (0.83)
Physical health	54.13 (6.30)	53.05 (7.10)	53.28 (6.94)
Mental health	41.26 (13.83)	38.40 (13.27)	39.01 (13.42)
Exercise *	12.63 (5.03)	9.92 (5.22)	10.50 (5.29)
Stress *	3.81 (1.22)	3.22 (1.14)	3.35 (1.18)
Diet	6.96 (2.77)	6.98 (3.29)	6.98 (3.18)

Note: *t* tests for independent samples were used to compare differences between men and women. * Significant differences between men and women (*p* < 0.05).

**Table 2:** Sleep quality and physical health (outcome variable: physical health).

		Coefficient	95% Confident Interval		*p*	Beta
Global Sleep Quality (unadjusted model)		−0.362	−0.590	−0.133	0.002	−0.172
Global Sleep Quality (adjusted model)		−0.290	−0.522	−0.057	0.015	−0.138
Subjective Sleep Quality sub-scale (unadjusted model; reference category: fairly good)						
	good	−0.156	−2.461	2.149	0.894	−0.011
	bad	−2.459	−4.967	0.049	0.055	−0.159
	very bad	−6.502	−10.644	−2.360	0.002	−0.187
Subjective Sleep Quality sub-scale (adjusted model; reference category: fairly good)						
	good	0.306	−2.008	2.620	0.795	0.022
	bad	−1.769	−4.311	0.773	0.172	−0.114
	very bad	−4.968	−9.170	−0.766	0.021	−0.143
Sleep Latency (unadjusted model)		−0.011	−0.038	0.015	0.396	−0.046
Sleep Latency (adjusted model)		−0.009	−0.035	0.017	0.501	−0.036
						
Sleep Duration (unadjusted model)		0.393	−0.153	0.938	0.158	0.078
Sleep Duration (adjusted model)		0.395	−0.158	0.948	0.161	0.079
						
Sleep Efficiency (unadjusted model)		−0.004	−0.041	0.034	0.854	−0.010
Sleep Efficiency (adjusted model)		−0.007	−0.044	0.030	0.707	−0.021
						
Sleep Disturbances (unadjusted model)		−0.360	−0.567	−0.154	0.001	−0.184
Sleep Disturbances (adjusted model)		−0.322	−0.528	−0.117	0.002	−0.165
Use of sleeping medication (unadjusted model; reference category: not during the past month)						
	Less than once a week	0.212	−2.239	2.662	0.865	0.009
	Once or twice a week	−2.269	−6.468	1.930	0.289	−0.058
	Three or more times a week	−2.645	−6.266	0.976	0.152	−0.079
Use of sleeping medication (adjusted model; reference category: not during the past month)						
	Less than once a week	0.056	−2.360	2.473	0.963	0.002
	Once or twice a week	−1.399	−5.586	2.789	0.512	−0.036
	Three or more times a week	−2.482	−6.026	1.062	0.169	−0.074
Day time dysfunction (unadjusted model; reference category: 0 points)						
	1 or 2 points	1.073	−1.011	3.158	0.312	0.077
	3 or 4 points	−1.154	−3.333	1.025	0.298	−0.078
	5 or 6 points	−1.117	−4.501	2.267	0.517	−0.040
Day time dysfunction (adjusted model; reference category: 0 points)						
	1 or 2 points	1.103	−0.943	3.149	0.290	0.079
	3 or 4 points	−0.706	−2.863	1.450	0.520	−0.048
	5 or 6 points	−0.475	−3.936	2.986	0.787	−0.017

Note: PSQI: Pittsburgh Sleep Quality Index; Adjusted models include: age, sex, diet, stress and exercise as covariates; Beta: standardized regression coefficient.

**Table 3:** Sleep quality and mental health (outcome variable: mental health).

		Coefficient	95% Confident Interval		*p*	Beta
Global Sleep Quality (unadjusted model)		−1.674	−2.084	−1.264	<0.001	−0.410
Global Sleep Quality (adjusted model)		−1.419	−1.835	−1.002	<0.001	−0.348
Subjective Sleep Quality sub-scale (unadjusted model; reference category: fairly good)						
	good	−5.132	−9.490	−0.773	0.021	−0.190
	bad	−11.122	−15.865	−6.380	<0.001	−0.371
	very bad	−16.377	−24.208	−8.546	<0.001	−0.244
Subjective Sleep Quality sub-scale (adjusted model; reference category: fairly good)						
	good	−3.040	−7.336	1.255	0.165	−0.113
	bad	−8.064	−12.783	−3.346	0.001	−0.269
	very bad	−11.937	−19.738	−4.136	0.003	−0.178
Sleep Latency (unadjusted model)		−0.030	−0.081	0.021	0.248	−0.063
Sleep Latency (adjusted model)		−0.023	−0.072	0.025	0.349	−0.049
						
Sleep Duration (unadjusted model)		1.829	0.794	2.865	0.001	0.189
Sleep Duration (adjusted model)		1.253	0.220	2.286	0.018	0.129
						
Sleep Efficiency (unadjusted model)		0.112	0.041	0.183	0.002	0.170
Sleep Efficiency (adjusted model)		0.090	0.022	0.159	0.010	0.136
						
Sleep Disturbances (unadjusted model)		−1.257	−1.640	−0.874	<0.001	−0.333
Sleep Disturbances (adjusted model)		−1.092	−1.464	−0.719	<0.001	−0.289
Use of sleeping medication (unadjusted model; reference category: not during the past month)						
	Less than once a week	−4.601	−9.232	0.029	0.051	−0.105
	Once or twice a week	−14.231	−22.166	−6.297	<0.001	−0.189
	Three or more times a week	−7.153	−13.994	−0.311	0.041	−0.110
Use of sleeping medication (adjusted model; reference category: not during the past month)						
	Less than once a week	−4.226	−8.684	0.232	0.063	−0.096
	Once or twice a week	−10.792	−18.517	−3.066	0.006	−0.143
	Three or more times a week	−6.536	−13.075	0.003	0.050	−0.101
						
Day time dysfunction (unadjusted model; reference category: 0 points)						
	1 or 2 points	−6.338	−9.955	−2.720	0.001	−0.234
	3 or 4 points	−12.480	−16.262	−8.698	<0.001	−0.437
	5 or 6 points	−24.964	−30.837	−19.090	<0.001	−0.460
Day time dysfunction (adjusted model; reference category: 0 points)						
	1 or 2 points	−6.060	−9.587	−2.533	0.001	−0.224
	3 or 4 points	−11.363	−15.081	−7.647	<0.001	−0.398
	5 or 6 points	−21.550	−27.515	−15.584	<0.001	−0.397

Note: PSQI: Pittsburgh Sleep Quality Index; Adjusted models include: age, sex, diet, stress and exercise as covariates; Beta: standardized regression coefficient.

## Data Availability

Data available on request due to restrictions.
